# New Zealand's Food System Is Unsustainable: A Survey of the Divergent Attitudes of Agriculture, Environment, and Health Sector Professionals Towards Eating Guidelines

**DOI:** 10.3389/fnut.2019.00099

**Published:** 2019-07-16

**Authors:** Rebekah Jones, Carol Wham, Barbara Burlingame

**Affiliations:** ^1^School of Sport, Exercise and Nutrition, Massey University, Auckland, New Zealand; ^2^School of Health Sciences, Massey University, Wellington, New Zealand

**Keywords:** sustainability, attitudes, food-based dietary guidelines, agriculture, environment, health, professionals, sectoral

## Abstract

**Background:** The Food and Agriculture Organisation has called for sustainable diets, which align with SDG 2, Zero Hunger, and SDG 12, Sustainable Consumption and Production. The inclusion of sustainability characteristics in New Zealand's (NZ) eating and activity guidelines (EAGs) may lead to achieving sustainable diets. This study aimed to evaluate the agreement among sectoral professionals of including sustainability characteristics within the guidelines.

**Methods:** Agriculture, environment, and health sector professionals were invited to complete an online survey to establish agreement with sustainability characteristics and sustainability statements. Opinion and attitude questions were completed using a 5-item Likert scale. One-way ANOVA analyses were conducted to compare the level of agreement and differences in means of the sector levels of agreement whilst controlling for covariates. *Post-hoc* tests were used to determine sectoral differences.

**Results:** Overall, 298 (65% female) respondents completed the survey from the agriculture (37%), environment (22%), and health (41%) sectors. Two-thirds (66%) of respondents were over 35 years and 90% had a tertiary education. Two-thirds (63%) of respondents disagreed that NZ's current food system is sustainable; health (77%) and environment (78%) sector respondents had greater disagreement than those from agriculture (35%; *P* = 0.00). Overall, 77% of respondents agreed that sustainability characteristics should be included in guidelines; health (90%) and environment (84%) sector respondents had greater agreement than from agriculture (58%; *P* = 0.00). Five sustainability characteristics received high levels of agreement (>90%) for inclusion: dietary diversity, sustainable seafood, limit processed foods, reduced food waste, and sustainable lifestyle behaviours. Agreement for eight sustainability characteristics was highest among the health and environment sectors vs. the agricultural sector (*P* < 0.05). A relatively low level of agreement was received from all three sectors, particularly the environmental sector (68.7%), towards the characteristic “to consume recommended serves of dairy products.” Only 38.5% of all respondents agreed with the inclusion of “organic food produce.” Negative associations were observed between respondents' opinions regarding the sustainability of NZ's current food system and familiarity with the EAGs.

**Conclusion:** Professionals from the agriculture, environment, and health sectors largely support the inclusion of sustainability characteristics in NZ's EAGs. A multi-sectoral approach will be required to address areas of divergence.

## Introduction

The global food system faces an ambitious challenge in meeting nutritional demands whilst reducing negative environmental impacts (1–3). Several initiatives have elaborated the concept of sustainable diets as the key for linking nutrition and sustainable food systems (4–9).

Sustainable diets have been defined as those diets with low environmental impacts which contribute to food and nutrition security and to healthy life for present and future generations. Sustainable diets are “protective and respectful of biodiversity and ecosystems, culturally acceptable, accessible, economically fair, and affordable; nutritionally adequate, safe and healthy while optimising natural and human resources” (10).

The importance of sustainable diets is further reflected in the 17 UN Sustainable Development Goals (SDGs); in particular, Goal 2 (“end hunger, achieve food security and improved nutrition, and promote sustainable agriculture”) and Goal 12 (“responsible production and consumption”) (11).

A wealth of literature suggests that no country currently meets basic dietary needs for its citizens at a globally sustainable level of resource use (12–14). Human dietary patterns, both current and emerging, threaten human and environmental health (4–6, 8, 15, 16). The production and consumption of highly processed and animal-based foods, as well as inadequate fruit, vegetable, fibre, and essential micronutrient intake, have resulted in human and planetary health degradation (17).

As per other developed nations, New Zealand (NZ) is experiencing growing rates of obesity and associated non-communicable diseases (18, 19). These contribute a significant health burden on individuals, families and the nation. Further, although NZ is a small emitter in absolute terms, accounting for <0.2% of global emissions, its food system is a major contributor to climate change, with per-capita emissions fifth highest among Organisation for Economic Co-operation and Development (OECD) countries in 2011 (20). The environmental impact of the food system is evident in NZ: damaged ecosystems, depleted fish stocks, soil degradation and loss of biodiversity, with more change expected (2, 21–23).

In response, nutrition and sustainability are high priority on the global political agenda (24–26). Research has highlighted that, while supply-end strategies, when implemented widely, have the potential to lead to important emissions reductions (6), the potential to reduce emissions arising from within the supply chain is not nearly as significant as those that can be achieved through a demand-end approach: namely, decreasing consumption of those agricultural products that are GHG-intensive (8, 22). According to a recent report by the Food Climate Research Network, based at Oxford University, a country's national dietary guidelines represent a key opportunity for policy development to address consumption patterns (27). Further, with international public interest in the link between nutrition, the environment and the food system increasing, this development may provide a greater incentive for the public to follow the recommendations and provide the government with an opportunity to take a progressive stance on this issue (3, 26, 28).

A range of possible sustainable diets exist, made up of several sustainable characteristics, each of which contribute to human and planetary health (7, 9, 29). Characteristics are features of a sustainable diet such as the consumption of local produce (7, 9, 10, 26, 30, 31). Donini et al. (30) define a suite of the most appropriate nutrition and health indicators for assessing the sustainability of diets based on the traditional Mediterranean diet. These include the biochemical characteristics of food including vegetable/animal protein consumption ratios, dietary energy adequacy, dietary energy density score, and nutrient density of diet; food quality including fruit and vegetable consumption and dietary diversity; environmental factors including food biodiversity composition and consumption, rate of local/regional foods, seasonality and eco-friendly food production and/or consumption; and lifestyle factors including physical activity/physical inactivity prevalence and adherence to the Mediterranean dietary pattern. Clinical aspects including diet-related morbidity/mortality statistics and nutritional anthropometry are also included (30).

A second reference for sustainable dietary characteristics has been provided by the EAT-Lancet Commission (31). They present a reference global planetary health diet that is healthy for both people and planet. This provides a basis for estimating the health and environmental effects of adopting an alternative diet to standard current diets (31). The final report recommends that a planetary health diet consists of vegetables, fruits, whole grains, legumes, nuts, and unsaturated oils. Further, it includes a low to moderate amount of seafood and poultry and no, or a low quantity of, red meat, processed meat, added sugar, refined grains, and starchy vegetables. Protein should be sourced from plants as much as possible and fish or alternatives sources of omega-3 fatty acids should be included several times per week (196 g/week). In addition, dairy consumption is optional, however if consumed, moderate levels of around 250 g/day are recommended (31).

Thirdly, overarching sustainability characteristics, for potential inclusion in food-based dietary guidelines (FBDGs), have been identified based on their inclusion in international sustainable FBDGs (32). These dietary guidelines integrate health and environmental sustainability considerations through the inclusion of some or all of the following recommendations; increase dietary biodiversity, consume a plant-based diets, moderate/limit red meat consumption, limit processed meat consumption, moderate dairy consumption, encourage sustainable seafood consumption, limit processed and ultra-processed foods, promote water conservation in cooking, promotion of buying local and seasonal foods, encouraging food and packaging waste reduction, sustainable behaviours including exercise and cooking at home and ethical animal welfare promotion. These sustainability characteristics all contribute to both human and planetary health (26).

Introducing these characteristics into national dietary guidelines will allow governments to act on nutrition-related health objectives, while concomitantly addressing sustainability concerns (6). Internationally, governments are beginning to include sustainability characteristics into their national dietary guidelines but are moving at different rates. There are now at least 12 countries which include the promotion of one or more of the sustainable diet characteristics into dietary guidelines (26), including Brazil, Qatar, Sweden and more recently, Canada (2019). These examples integrate sustainability throughout the entire document as a primary, interconnected consideration for healthy eating.

The food system is complex. Several social, economic, and political interactions exist between people and their food. Consequently, development and implementation of meaningful food-based dietary guidelines (FBDGs) has been associated with many challenges. The recognition of the need to integrate the full suite of impacts that dietary choices and the food system can have on the environment has exacerbated these challenges (6, 26, 33–37).

Although health, environmental, and humanitarian concerns are common to all sectors, differences lie in the degree to which each sector, and professionals within these sectors, perceive the importance of and viability of methods for addressing these issues as they try to achieve their own set of interests (38). The premise of a widespread dietary shift toward greater incorporation of plant-based foods and a reduction in animal-based products is viewed as “drastic and unrealistic” by many stakeholders (39).

Many stakeholders have been seen to use their political influence to block or reverse policies that would make the food system more sustainable. For example, scientific committees in both the US and Australia have attempted to include environmental considerations in their respective FBDGs (40). However, due to a lack of government endorsement, resistance and lobbying against their implementation from a range of sectors and bodies, the most recent revisions of guidelines do not explicitly include sustainability characteristics.

In contrast, some governments have begun to incorporate a wider range of expertise and representation in their consultation and development processes of FBDGs with sustainability characteristics. For example, the 2014 Brazilian FBDGs have included representation from the education, social welfare and, agriculture sectors, as well as the public (41). This strategy has ensured that the broader societal and environmental issues of the food system are addressed and included in the development and implementation processes. By including multiple perspectives in the development process, Brazil ensured that all recommendations were translated in a way that could be understood and followed by all stakeholders. This process has helped to mitigate resistance, lobbying and abandonment and allowed for successfully developing and implementing FBDGs with sustainability at the forefront, supported by all sectors.

NZ's 2015 *Eating and Activity Guidelines for Adults* do not explicitly include sustainability recommendations (19, 26). However, when the findings of New Zealand's Adult Nutrition Survey 08/09 and the New Zealand Health Survey 2017/18 are compared directly to the nutrition indicators of sustainability proposed by Donini et al. (30) and the EAT-Lancet Commission's healthy reference diet, we find several discrepancies (18, 19, 31, 42). These discrepancies suggest that NZ's current food system and diets are unsustainable. This provides an opportunity to address sustainability issues via FBDGs.

In response, Drew et al. (28) has conducted a contextual analysis toward developing sustainability considerations, specifically, for inclusion within NZ's dietary guidelines. It is suggested that a diet consisting of whole plant foods, including vegetables, fruits, legumes, and whole grains, is found to be far less emissions-intensive than a diet consisting of mostly animal-based foods, particularly red and processed meats, in line with international literature.

As an essential and integral component of NZ's national food policy, the inclusion of sustainability characteristics within the new dietary guidelines is plausible. However, from international examples of sustainable FBDG implementation, it is evident that systemic change is required to address the most pressing sustainability issues. Achieving this requires interdisciplinary collaboration from academia, government and industry stakeholders prior to implementation.

Given that NZ's food system is a primary driver of detrimental change to the environment and that the burden of nutrition-related chronic disease continues to grow around the world, the importance of focusing research and policy efforts on healthy and sustainable eating patterns is incontestable (28, 43).

However, it is still unclear how such a focus would be received by key stakeholders in NZ. Currently, there is no local research specifically reporting sectoral professionals' attitudes or opinions towards proposed, or implemented, FBDG sustainability recommendations.

Thus, the purpose of this study was to provide a quantitative account of sectoral professionals' opinions and attitudes towards the inclusion of sustainability characteristics in NZ's EAG series prior to implementation. Convergence of agreement between sectoral groups towards the inclusion of individual sustainability characteristics within NZ's EAGs will be examined.

## Methods

### Purpose

The purpose of this study was to evaluate the divergence and convergence of agreement among sectoral groups towards the inclusion of identified sustainability characteristics within New Zealand's eating and activity guidelines (EAGs). Attitude and opinion were assessed through a series of Likert scale questions (44–46). The primary hypothesis of this study was that there are sectoral biases, and that sectoral professionals will not converge in their level of agreement about the inclusion of sustainability characteristics with NZ EAGs. International experience suggests that sectoral professionals do not agree with the inclusion of some sustainability characteristics within food-based dietary guidelines as evidenced by lobbying and subsequent abandonment of sustainable FBDGs (26, 40, 47, 48).

### Participants

Professionals from the health, environment and agriculture sectors of NZ over 18 years of age were invited to complete an online survey through contact with governing bodies, professional associations, industry associations, and advocacy groups within the health, environmental and agricultural sectors. Gender, age, education, and professional sector were determined. Participants provided consent to the terms of the study at the initiation of the survey.

### Survey Design: Attitude and Opinion

Opinions of the sustainability of NZ's current food system, and the current status of NZ's EAGs were measured in this survey using sustainability statements. Attitudes towards the inclusion of individual sustainability characteristics within NZ's EAGs were measured.

Each questionnaire item asked participants to state their level of agreement with either a sustainability statement or their agreement with the inclusion of a sustainability characteristic in NZ's FBDGs. The characteristics included in the development of the questionnaire were based on indicators defined by Donini et al. (30) and the EAT-Lancet Commission (31) as well as sustainability statements included in international sustainable FBDGs (32). Each sustainability statement and characteristic were written using plain English and avoided jargon or any technical term that was outside of common usage. The opinion and attitude questions were completed using a 5-item Likert scale ranging from strongly agree, agree, don't know, disagree, to strongly disagree. The survey comprised three sections; demographic characteristics of the participants, sustainability statements, and individual sustainability characteristics to discourage pattern answering (49).

### Pre-testing the Questionnaire

To gain insight into whether survey questions were understandable, logical, and understood in the manner they were intended, a pilot study was undertaken prior to the final survey being released including feedback from Ministry of Health key informants. The data gathered with the online pilot survey were tested to ensure appropriate data analyses were selected as well as logical progression and accessibility of the survey for the participants. For the most part, the pilot survey was deemed too long, which led to a consolidation of questions and removal of lengthy explanations of individual sustainability characteristics. For the full survey used in the research, refer to Appendix A.

### Sample Size

Based on NZ Business Demography Statistics there was a 120,700 Employee Count in the Agriculture, Forestry and Fishing sector and 227,000 in the Health Care and Social Assistance sector. This indicates the total population of agriculture, environmental and health professionals in New Zealand is in excess of 300,000 (50). Therefore, to conduct analyses of covariance with these three sector groups, regardless of the relative size of the sectors, an estimated total of 271 participants was determined. This calculation was based on a 300,000-population size with a 90% confidence interval, and a 5% margin of error (51, 52).

No population statistics were available through Statistics New Zealand for the environmental sector. However, as the sample count of the Agriculture and Health sectors is in excess of 300,000 the number of employees in the environment sector would not change the required total sample size (51, 52).

### Procedure

Surveys were distributed to participants via email, web, and social media links made available through Survey Monkey (Survey Monkey Inc, Palo Alto, CA, USA). The link was made available to the participants on Wednesday 20 June 2018. Emails were sent to 419 individuals, plus six governing bodies either posted the survey link on their websites or included it in their monthly newsletters. As this survey employed a “snowball” effect, it is impossible to say how many invitations to the survey were disseminated. A total of 302 responses were obtained via email (81 responses), web links (215 responses), and social media posts (6 responses).

On the survey closing date, collected responses were directly transferred to IBM SPSS 25 (2017) for analysis.

### Analysis

Validation and imputation of the data were completed by editing individual records. The sector (Health, Environment, or Agriculture) participants identified as working under, was used to identify professionals of interest to the survey. Respondents who selected their sector as “other” were edited by secondary validation; that is, their answer to the next question or previous response was used to determine which sector they belonged to. In addition, valuable subjective source, that is, the *Australian and New Zealand Standard Industrial Classification (ANZSIC)* (2006) was used to categorise responses into the correct sector. The most common use for this was respondents who selected “Other” then specified “Horticulture” in the comment box which, for the purposes of this study, falls under the “Agriculture” sector. An additional sub-sector group was created, labelled “Agriculture—other” for respondents who identified as working for more than one agricultural sub-sector or one that was not an available option, such as “bees.” Considering respondents' sector while comparing it to their level of agreement with each sustainability characteristic provided an opportunity to use parametric statistics.

One-way ANOVA analyses were conducted to compare the level of agreement with the inclusion of sustainability statements of the sector groups: agriculture, environment and health and demographic characteristics; age and education. As described in Appendix B, Table 1, results from a one-way ANOVA show that there is a statistically significant difference between age and education groups with level of agreement towards the inclusion of sustainability characteristic statements. Therefore, a one-way ANCOVA analysis was undertaken to detect a difference in level of agreement with the inclusion of sustainability statements of the sector groups: agriculture, environment and health means of the sector levels of agreement whilst controlling for covariates (Table 4). *Post-hoc* tests were used to determine specifically where the significant differences in opinion lay between the sectors.

**Table 1:** Demographic characteristics of respondents by sector.

	**Total**	**Sector (%)**		
		**Agriculture**	**Environment**	**Health**
	298 (100)	110 (37)	67 (22)	121 (41)
**GENDER**				
Men	35.2	57.3	41.4	9.9
Women	64.8	42.7	58.6	90.1
**AGE GROUP (YEARS)**				
18–24	5.0	0.0	4.5	10.0
25–34	28.5	15.5	35.8	36.7
35–44	23.8	22.7	25.4	24.2
45–54	19.1	25.5	22.4	11.7
55–64	17.9	23.6	10.4	15.8
65+	5.7	12.7	1.5	1.6
**EDUCATION**				
Secondary	10.8	24.5	6.0	0.8
Tertiary (Under-graduate)	28.5	40.9	29.8	15.7
Tertiary (Post-graduate)	60.7	34.6	64.2	83.5

A correlation analysis was then conducted between level of agreement responses to the inclusion of individual sustainability characteristics and level of respondents' agreement with sustainability statements; The world's current food system is sustainable, New Zealand's current food system is sustainable, New Zealand needs to adopt more/better agro-ecological farming practices, I am familiar with the 2015 “Eating and Activity Guidelines for New Zealand Adults” (*r* < 0.05^*^, *r* < 0.01^**^). This was done to estimate the association between respondents' opinions regarding the sustainability statements and their level of agreement with individual sustainability characteristics.

Following analyses, participant responses were collapsed into Disagree (D–Strongly Disagree and Disagree), Don't Know (DK), and Agree (A–Agree and Strongly Agree) for simplicity of presentation.

All data analyses were based only on non-missing data. Statistical significance was achieved if the *P*-value was < 0.05.

### Ethics

This project has been evaluated by peer review and judged to be low risk. Consequently, it has not been reviewed by one of the University's Human Ethics Committees. The researcher(s) named in this document are responsible for the ethical conduct of this research.

## Results

Following data cleaning, five respondents' surveys could not be used as they did not meet the target criteria, that is, their responses could not be clearly sorted into one of the specified sectors; agriculture, environment or health. The demographic characteristics of the participants are shown in Table 1. Of 298 respondents, approximately two-thirds were women. Most respondents were from the health sector (41%), a third (37%) were from the agricultural sector and 22% from the environmental sector. Most respondents (89.3%) were aged between 25 and 64 years. Within this, respondents were in the age brackets 25–34 years (28.5%), 35–44 years (23.8%), 45–54 years (19.1%), and 55–64 years (17.1%).

Most respondents (89.2%) had tertiary education and 60.7% had post-graduate education. Nearly all respondents from the health (99.2%) and environmental (94%) sectors had a tertiary education vs. three-quarters of those from the agriculture sector (75.5%). There were 11% of respondents with a high school education.

### Agreement With Sustainability Statements

As shown in Table 2, the majority of respondents (78.3%) do not believe the world's current food system is sustainable, with the lowest level of agreement by the agriculture sector (60%).

**Table 2:** Respondent agreement with sustainability statements and characteristics by sector.

**Sustainability statement**		**A (%)**	**DK (%)**	**D (%)**
The world's current food system is sustainable	Agriculture	27.3	12.7	60.0
	Environment	7.5	6.0	86.6
	Health	6.6	5.0	88.4
	Total	13.8	7.9	78.3
New Zealand's current food system is sustainable	Agriculture	57.4	7.4	35.2
	Environment	11.9	10.4	77.6
	Health	14.9	8.3	76.9
	Total	28	8.7	63.3
New Zealand needs to adopt more/better agro-ecological farming practices	Agriculture	80.9	5.5	13.6
	Environment	97.0	0.0	3.0
	Health	87.6	9.9	2.5
	Total	88.6	5.1	6.3
I am familiar with the 2015 “Eating and Activity Guidelines for New Zealand Adults”	Agriculture	34.5	30.9	34.5
	Environment	13.6	24.2	62.1
	Health	90.9	1.7	7.4
	Total	46.3	19	34.7
Sustainability recommendations should be included in the “Eating and Activity Guidelines for Adults”	Agriculture	57.4	7.4	35.2
	Environment	83.6	10.4	6.0
	Health	90.0	5.0	5.0
	Total	77	7.6	15.4
**SUSTAINABILITY CHARACTERISTIC**				
Promotion of diet diversity/variety of whole foods	Agriculture	97.2	1.8	0.9
	Environment	95.5	3.0	1.5
	Health	98.3	0.8	0.8
	Total	97	1.9	1.1
Promotion of plant-based diets	Agriculture	42.7	13.6	43.6
	Environment	77.6	9.0	13.4
	Health	87.6	5.0	7.4
	Total	68.3	9.2	21.4
To limit red meat consumption as per recommendations	Agriculture	50.9	15.5	33.6
	Environment	86.4	6.1	7.6
	Health	90.0	5.8	4.2
	Total	75.8	9.1	15.1
To limit processed meat consumption as per recommendations	Agriculture	78.2	7.3	14.5
	Environment	95.5	3.0	1.5
	Health	99.2	0.8	0.0
	Total	91	3.7	5.3
To consume recommended serves of dairy products	Agriculture	73.6	11.8	14.5
	Environment	68.7	14.9	16.4
	Health	80.8	7.5	11.7
	Total	74.4	11.4	14.2
Promotion of sustainable seafood consumption	Agriculture	87.3	5.5	7.3
	Environment	91.0	7.5	1.5
	Health	94.2	1.7	4.2
	Total	90.8	4.9	4.3
To limit/reduce ALL processed foods high in fat, salt, and sugar as per recommendations	Agriculture	87.2	8.3	4.6
	Environment	88.1	7.5	4.5
	Health	96.7	1.7	1.7
	Total	90.7	5.8	3.6
To purchase and support local food produce	Agriculture	74.3	4.6	21.1
	Environment	88.1	6.0	6.0
	Health	92.6	4.1	3.3
	Total	85	4.9	10.1
To purchase and support seasonal food produce	Agriculture	86.4	2.7	10.9
	Environment	97.0	3.0	0.0
	Health	97.5	0.0	2.5
	Total	93.6	1.9	4.5
To purchase and support organic food produce	Agriculture	21.8	16.4	61.8
	Environment	59.7	14.9	25.4
	Health	33.9	24.0	42.1
	Total	38.5	18.4	43.1
Standards for the ethical treatment of livestock	Agriculture	87.3	2.7	10.0
	Environment	94.0	4.5	1.5
	Health	80.8	8.3	10.8
	Total	87.4	5.2	7.4
To reduce food waste	Agriculture	93.6	2.7	3.6
	Environment	94.0	4.5	1.5
	Health	98.3	0.8	0.8
	Total	95.3	2.7	2.0
Promotion of sustainable lifestyle behaviours (for example, physical activity)	Agriculture	96.4	3.6	0.0
	Environment	97.0	3.0	0.0
	Health	98.3	1.7	0.0
	Total	97.2	2.8	0.0
**SUSTAINABILITY STATEMENT**				
I support country of origin labelling of foods	Agriculture	89.2	2.0	8.8
	Environment	93.4	4.9	1.6
	Health	97.3	2.7	0.0
	Total	93.3	3.2	3.5
I support labelling foods with New Zealand Geographic Indicators	Agriculture	75.2	9.2	15.6
	Environment	85.1	11.9	3.0
	Health	81.0	12.4	6.6
	Total	80.4	11.1	8.4

^*^Introductory statement for question 7 of survey; “The following characteristics of a sustainable diet should be included and linked to both human and environmental health in the ‘Eating and Activity Guidelines for New Zealand Adults’:”

*Original scale: 1 = Strongly Disagree (SD), 2 = Disagree (D), 3 = Neither Agree or Disagree (N), 4 = Agree (A), 5 = Strongly Agree (A). For simplicity of presentation “Strongly Disagree” and ‘Disagree’ (1+2) have been collapsed to ‘Disagree’ (D) and ‘Agree’ and ‘Strongly Agree’ (4+5) have been collapsed to Agree (A)*.

Overall, there is 77% agreement from all sectoral professionals that sustainability recommendations should be included in the eating and activity guidelines (EAGs) for adults in NZ.

Two-thirds of respondents (63.3%) believe NZ's current food system is not sustainable. Most respondents (88.6%) believe that NZ needs to adopt more/better agro-ecological farming practices. Just under half of respondents were familiar with the 2015 *Eating and Activity Guidelines for New Zealand Adults* (46.3%).

### Agreement With Inclusion of Individual Sustainability Characteristics

Tables 3, 4 indicate five of the sustainability characteristics received unanimously high levels of agreement for inclusion in New Zealand's EAGs from all three sectors (>90%); namely, promotion of dietary diversity (97%), sustainable seafood (90.8%), to limit processed foods (90.7%), reduction of food waste (95.3%), and promotion of sustainable lifestyle behaviours (97.2%).

**Table 3:** Respondent agreement with sustainability statements and characteristics by sector.

**Sustainability statement**	**Sector**	**Mean (SD)**	**Total sample mean (SD)**	***P*-value**
The world's current food system is sustainable	Agriculture	3.47 (1.1)	3.93 (1.0)	0.00*
	Environment	4.16 (0.9)		
	Health	4.21 (0.8)		
New Zealand's current food system is sustainable	Agriculture	2.66 (1.2)	3.42 (1.2)	0.00*
	Environment	3.88 (1.0)		
	Health	3.84 (0.9)		
New Zealand needs to adopt more/better agro-ecological farming practices	Agriculture	2.15 (0.9)	1.82 (0.857)	0.003*
	Environment	1.43 (0.7)		
	Health	1.173 (0.7)		
I am familiar with the 2015 “Eating and Activity Guidelines for New Zealand Adults”	Agriculture	3.06 (1.2)		0.00*
	Environment	3.70 (1.1)	2.62 (1.358)	
	Health	1.62 (0.9)		
Sustainability recommendations should be included in the “Eating and Activity Guidelines for Adults”	Agriculture	2.68 (1.1)		0.00*
	Environment	1.96 (0.8)	2.11 (1.024)	
	Health	1.67 (0.8)		
**Sustainability characteristic**		**A (%)**	**DK (%)**	**D (%)**
Promotion of diet diversity/variety of whole foods	Agriculture	1.58(0.6)		0.627
	Environment	1.66(0.7)		
	Health	1.33(0.6)	1.49 (0.621)	
Promotion of plant-based diets	Agriculture	3.12 (1.3)		0.00*
	Environment	2.07 (1.0)		
	Health	1.77 (0.9)	2.34 (1.204)	
To limit red meat consumption as per recommendations	Agriculture	2.83 (1.1)	2.15 (1.085)	0.00*
	Environment	1.83 (0.9)		
	Health	1.70 (0.8)		
To limit processed meat consumption as per recommendations	Agriculture	2.14 (1.0)		0.00*
	Environment	1.52 (0.6)	1.66 (0.826)	
	Health	1.01 (0.1)		
To consume recommended serves of dairy products	Agriculture	2.32 (0.9)		0.272
	Environment	2.37 (1.1)	2.23 (1.026)	
	Health	2.06 (1.1)		
Promotion of sustainable seafood consumption	Agriculture	1.84 (0.8)		0.209
	Environment	1.61 (0.7)	1.68 (0.806)	
	Health	1.58 (0.8)		
To limit/reduce ALL processed foods high in fat, salt, and sugar as per recommendations	Agriculture	1.81 (0.8)		0.05
	Environment	1.66 (0.9)	1.62 (0.780)	
	Health	1.42 (0.6)		
To purchase and support local food produce	Agriculture	2.10 (1.3)	1.79 (1.036)	0.00*
	Environment	1.75 (0.9)		
	Health	1.53 (0.8)		
To purchase and support seasonal food produce	Agriculture	1.81 (1.0)	1.54 (0.817)	0.001*
	Environment	1.51 (0.6)		
	Health	1.31 (0.6)		
To purchase and support organic food produce	Agriculture	3.55 (1.3)		0.00*
	Environment	2.43 (1.2)	3.09 (1.293)	
	Health	3.04 (1.2)		
Standards for the ethical treatment of livestock	Agriculture	1.87 (1.0)	1.80 (0.946)	0.040*
	Environment	1.55 (0.7)		
	Health	3.04 (1.2)		
To reduce food waste	Agriculture	1.53 (0.8)	1.37 (0.664)	0.188
	Environment	1.33 (0.6)		
	Health	1.24 (0.5)		
Promotion of sustainable lifestyle behaviours (for example, physical activity)	Agriculture	1.52 (0.6)		0.641
	Environment	1.40 (0.6)	1.38 (0.539)	
	Health	1.24 (0.5)		
**Sustainability statement**		**A (%)**	**DK (%)**	**D (%)**
I support country of origin labelling of foods	Agriculture	1.70 (1.0)		0.008*
	Environment	1.41 (0.7)	1.50 (0.785)	
	Health	1.37 (0.5)		
I support labelling foods with New Zealand Geographic Indicators	Agriculture	2.09 (1.093)		0.05
	Environment	1.70 (0.789)	1.93 (0.947)	
	Health	1.91 (0.856)		

* *Introductory statement for question 7 of survey; “The following characteristics of a sustainable diet should be included and linked to both human and environmental health in the ‘Eating and Activity Guidelines for New Zealand Adults’:*”

^*^ Identifies level of significance P < 0.05. Original scale analysed: 1 = Strongly Disagree (SD), 2 = Disagree (D), 3 = Neither Agree or Disagree (N), 4 = Agree (A), 5 = Strongly Agree (A).

**Table 4:** Respondent agreement with sustainability statements and characteristics with statistically significant difference (*P* < 0.05) by sector controlling for gender, age, and education.

			**ANCOVA covariate *P*-value**			
**Sustainability statement**		***P-*value**	**Sector**	**Gender**	**Age (years)**	**Education**
The world's current food system is sustainable	Agriculture^ab^	0.00*	0.005*	0.102	0.199	0.177
	Environment^a^					
	Health^b^					
New Zealand's current food system is sustainable	Agriculture^ab^	0.00*				
	Environment^a^		0.000*	0.706	0.994	0.616
	Health^b^					
New Zealand needs to adopt more/better agro-ecological farming practices	Agriculture^a^	0.003*				
	Environment^a^					
	Health^a^		0.007*	0.190	0.107	0.914
I am familiar with the 2015 “Eating and Activity Guidelines for New Zealand Adults”	Agriculture^a^	0.00*				
	Environment^a^				0.127	0.022*
	Health^a^		0.000*	0.224		
Sustainability recommendations should be included in the “Eating and Activity Guidelines for Adults”	Agriculture^ab^	0.00*				
	Environment^a^					
	Health^b^		0.000*	0.449	0.587	0.027*
**SUSTAINABILITY CHARACTERISTIC**						
Promotion of plant-based diets	Agriculture^ab^	0.00*				
	Environment^a^		0.000*	0.4698	0.938	0.010*
	Health^b^					
To limit red meat consumption as per recommendations	Agriculture^ab^	0.00*				
	Environment^a^		0.000*	0.422	0.840	0.006*
	Health^b^					
To limit processed meat consumption as per recommendations	Agriculture^ab^	0.00*				
	Environment^a^		0.001*	0.403	0.685	0.082
	Health^b^					
To purchase and support local food produce	Agriculture^ab^	0.00*				
	Environment^a^		0.000*	0.036*	0.100	0.000*
	Health^b^					
To purchase and support seasonal food produce	Agriculture^ab^	0.001*				
	Environment^a^			0.063	0.022*	0.011*
	Health^b^		0.000*			
To purchase and support organic food produce	Agriculture^ab^	0.00*				
	Environment^ab^		0.000*	0.019*	0.290	0.084
	Health^ab^					
Standards for the ethical treatment of livestock	Agriculture	0.040*	0.017*	0.111	0.085	0.027*
	Environment ^a^		0.000*	0.019*	0.290	0.084
	Health ^a^					
**SUSTAINABILITY STATEMENT**						
I support country of origin labelling of foods	Agriculture ^a^	0.008*	0.004*	0.235	0.565	0.006*
	Environment					
	Health ^a^					
I support labelling foods with New Zealand Geographic Indicators	Agriculture	0.05				
	Environment		0.074	0.626	0.982	0.425*
	Health					

* *Identifies level of significance P < 0.05. Values with the shared superscript represent significant differences according to the Tukey HSD post-hoc test (P < 0.05)*.

A significantly lower level of agreement from the agriculture sector was present for the promotion of seasonal food produce and standards for the ethical treatment of livestock; (*P* < 0.05), and for five sustainability characteristics; promotion of plants-based diets, limit red meat, limit processed meat, to purchase and support local produce and country of origin labelling of foods (*P* < 0.05).

The characteristic, “to consume recommended serves of dairy products,” received a relatively low level of agreement from all three sectors, with only three-quarters of all respondents (74.4%) agreeing with the inclusion of this recommendation. The lowest level of agreement for the dairy product item was from the environmental sector (68.7%) and highest level of agreement (80.8%) from the health sector.

Further, while 80.4% of all respondents agreed with the item, “I support labelling foods with New Zealand Geographic Indicators,” three-quarters (75.2%) of the agriculture sector respondents agreed.

Only 38.5% of all respondent agreed with the inclusion of the characteristic, “To purchase and support organic food produce”: agriculture (21.8%), environment (59.7%), and health (33.9%). *P* ≤ 0.01.

As shown in Table 5, of the eight individual sustainability characteristics which obtained a statistically significant difference in level of agreement between sectors, significant negative correlation exists between respondent's agreement with the sustainability statement “The World's current food system is sustainable” and their agreement with the inclusion of all of the sustainability characteristics except for “to purchase and support organic food produce” where there was a statistically significant positive correlation (*P* < 0.01).

**Table 5:** Correlation between significantly different levels of sector agreement towards sustainability characteristics and overall agreement with sustainability statements.

	**Sustainability statements**				
	**The World's current food system is sustainable**	**NZ's current food system is sustainable**	**New Zealand needs to adopt more/better agro-ecological farming practices**	**Sustainability recommendations should be included in the “Eating and Activity Guidelines for Adults”**	**I am familiar with the 2015 “Eating and Activity Guidelines for New Zealand Adults”**
**Sustainability characteristics**	*R*	*R*	*R*	*R*	*R*
Promotion of plant-based diets	−0.377**	−0.393**	0.952**	−0.245**	0.205**
To limit red meat consumption as per recommendations	−0.492**	−0.399**	0.980**	−0.377**	0.156**
To limit processed meat consumption as per recommendations	−0.645**	−0.298**	0.959**	−0.301**	0.130*
To purchase and support local food produce	−0.637**	−0.248**	0.921**	−0.298**	0.070
To purchase and support seasonal food produce	−0.650**	−0.232**	0.866**	−0.262**	0.053
To purchase and support organic food produce	0.649**	−0.279**	−0.208**	−0.574**	−0.098
Standards for the ethical treatment of livestock	−0.631**	−0.106	0.983**	−0.308**	−0.168**
I support country of origin labelling of foods	−0.675**	−0.193**	0.836**	−0.255**	0.085

* *Introductory statement for question 7 of survey; “The following characteristics of a sustainable diet should be included and linked to both human and environmental health in the ‘Eating and Activity Guidelines for New Zealand Adults’:*”

** *Correlation is significant at the 0.01 level (2-tailed)*.

^*^Correlation is significant at the 0.05 level (2-tailed).

A significant negative correlation exists between respondent's agreement with two of the sustainability statements “New Zealand's current food system is sustainable” and “Sustainability recommendations should be included in the ‘Eating and Activity Guidelines for Adults” their agreement with the inclusion of all of the sustainability characteristics (*P* < 0.01).

A significant positive relationship also exists between respondent's agreement with “New Zealand needs to adopt more/better agro-ecological farming practices” with all of the sustainability characteristics except agreement with “to purchase and support organic food produce” (*P* < 0.01).

Further, significant positive correlation was observed between respondents' familiarity with the 2015 *Eating and Activity Guidelines for New Zealand Adults* and four of the individual sustainability characteristics: promotion of plant-based diets, to limit red meat consumption as per recommendations, to limit processed meat, and standards for the ethical treatment of livestock (*P* < 0.01).

## Discussion

This is the first study, internationally, to assess the degree of convergence between sectoral groups for the inclusion of sustainability characteristics into national dietary guidelines. The focus of this study on New Zealand's Eating and Activity Guidelines (EAGs) will be of international interest as many of the advantages and challenges faced by New Zealand are similar to other developed countries. Overall, findings demonstrate strong support across NZ sectoral professionals, with 77% indicating agreement that sustainability recommendations should be included in the revised *Eating and Activity Guidelines for New Zealand Adults*.

This high level of support highlights the degree to which sectors view the importance of sustainable food systems as an important issue in NZ. The support demonstrated in this study, in line with international findings, suggests dietary guidelines are an appropriate medium for introducing sustainable eating patterns into nutrition policy and creating consumer awareness (5, 26, 53–55).

Similar multi-sectoral support has been demonstrated in Australia (36). Sectoral professionals have been responsive to the concept of combining health and sustainability, many already utilising it within their organisations. For example, as evidenced by the launch of One *Blue Dot: Environmentally Sustainable Diet Toolkit*, the British Dietetics Association's (BDA) Environmentally Sustainable Diet Project for dietitians (56, 57).

While this study's findings suggest the existence of overall support, the level of agreement varies by sector. Health professionals support the inclusion of sustainability recommendations within the EAGs the most (90%), followed by respondents from the environment (83.6%) and agriculture (57.4%) sectors.

Disunity between sectors has also been demonstrated in America, Australia, and Sweden (6, 32–37). These countries found that, due to variations in the individual sector's interests in the food system, high levels of overall support during development was later met with fierce opposition and subsequent abandonment of sustainability characteristics within FBDGs (6, 36). The divergence of opinion in the current study's findings therefore suggests a similar conflict may surround adoption of sustainable dietary guidelines in NZ.

In America, dietary guidelines are jointly developed and issued by the US Department of Health and Human Services as well as the US Department of Agriculture. In this case, the opposing opinions and subsequent lobbying of the agricultural sector carried enough influence during implementation to override the supporting views of health sector professionals. The administration structure, combined with economic influence, may explain the ongoing absence of sustainability characteristics in America. In contrast, successful development and implementation of national dietary guidelines with integrated sustainability principles in Qatar is thought to be primarily due to a lack of industry influence (58).

These examples, and the significantly lower level of support from the agriculture sector in the current study, posits the question whether primary industry should be part of the development of FBDGs. Suggestions have been made that industry should be omitted from dietary guideline development discussions and setting of the policy agenda, and only be involved in the implementation of actions (32, 59).

However, like NZ, Australian FBDG development is led solely by the health sector. Despite this administration structure, the widespread support for the integration of environmental considerations into guidelines was largely ignored in the latest revision (36). Again, the food and agriculture organisations and industry lobbyists in Australia were given disproportionate influence over the way that the National Food Plan was shaped (33).

The significantly lower level of support from the agriculture sector in this study highlights the need to mitigate the risk of sectoral lobbying in NZ via wider, earlier and higher levels of engagement with relevant sectors and individuals to increase overall multi-sectoral agreement, prior to implementation (60–62). Instead of excluding the primary industry from FBDG development and implementation, a number of changes, namely, to enhance transparency, manage biases, and conflicts of interest, may be required (63). By adopting this approach, FBDGs have been successfully developed and implemented in several countries including Brazil, Germany, Sweden, Netherlands, and Qatar, as well as into “quasi-official” guidelines in France and Estonia. Similar to NZ, guideline development is led by the health sector, but is elaborated in a participatory manner, in consultation with multiple sectors of the society, including agriculture and environment sectors (6, 26, 64, 65).

These examples suggest that the development of NZ guidelines can remain with the health sector but, as the agriculture sector's support for change is imperative, should be supported and guided by input from the agriculture and other relevant sectors (36, 57).

In order to understand the opinions and attitudes NZ sectoral professionals hold towards sustainable dietary guidelines, and to further explain the divergence of opinion between the sectoral groups, this study assessed each sector's level of agreement towards individual sustainability characteristics. It found an overall, unanimous, high level of agreement from professionals from the agriculture, environment, and health sectors with the inclusion of five of the 13 sustainability characteristics into the EAGs: promotion of dietary diversity (97%), sustainable seafood (90.8%), to limit processed foods (90.7%), reduction of food waste (95.3%), and promotion of sustainable lifestyle behaviours (97.2%; *P* > 0.05). Further, although a statistically significant difference of level of agreement was found between sectors (*P* < 0.05) for the inclusion of promotion of seasonal food produce (93.6%) and standards for the ethical treatment of livestock (87.4%), overall, a relatively high level of agreement from all sectors was shown. It is important to distinguish between convergence of opinion with an overall high level (%) of agreement, convergence of opinion with an overall low level of agreement and divergence with overall high level of agreement, when assessing whether opposition towards a sustainability characteristic is likely.

An overall high level of agreement, with or without convergence, suggests that the inclusion of these sustainability characteristics is likely to receive support if implemented into NZ's EAGs.

Worldwide, partial implementation of sustainability characteristics within FBDGs exists, tailored to the needs and challenges of each country (26). The results of the current study suggest that the sole inclusion of these seven characteristics may, at this time, act as a starting point for NZ towards implementing all sustainable dietary guidelines and should be included in the next revision of the EAGs currently taking place.

In contrast, divergence of opinion between sector groups, with a relatively lower level of agreement from one or more sectors, was associated with five of the thirteen proposed sustainability characteristics and one sustainability statement. Agreement with the promotion of plants-based diets, limit red meat, limit processed meat, to purchase and support local produce and inclusion of country of origin labelling of foods were all supported significantly less by the agriculture sector respondents, compared to the health and environment sector respondents (*P* < 0.05). Further, the item “to consume recommended serves of dairy products,” received a relatively low level of agreement from all three sectors with only three-quarters of all respondents (74.4%) agreeing with the inclusion of this item.

This divergence of opinion, as well as low level of agreement from specific sectoral groups towards these individual characteristics, may exist primarily due to vested interests associated with each of the sectors. It has been well-documented that vested interests, and conflict of interest, drive attitudes and subsequent behaviours (62, 66–68). Interests of sectoral groups, if in conflict with the proposed sustainability recommendations, may drive attitudes opposing the inclusion of sustainability characteristics into FBDGs. This may explain the abandonment of sustainability considerations in Australia's 2013 guideline revision, delayed implementation in Sweden and may be a barrier to the inclusion of sustainability characteristics in NZ's next EAG revision.

For example, both the health and environment sectors may be significantly more in favour of the inclusion of sustainability characteristics due to the close alignment of the characteristics with their goals. That is, that those diets that are good for human health are also healthy for the environment (69–72)

In contrast, dairy products have been shown to have significant negative environmental impacts. This may explain the significantly lower level of agreement from the environmental sector respondents (68.7%), suggesting they would prefer dairy consumption lower than the current guidelines. However, some dairy components, such as calcium, bioactive proteins, milk fatty acids, and the whole dairy food matrix, are considered indispensable sources of nutrition by the health sector. This may explain the health sector respondents' support of promoting dairy consumption to meet current health guidelines (80.8%), and their disagreement with reducing it further (73).

Many of the proposed sustainability characteristics for inclusion into NZ's EAGs may directly conflict with agricultural interests. Beef and lamb production are a key component of the NZ agriculture system but conflict with several proposed characteristics, such as reducing global livestock production, and the associated consumption of meat and dairy, which other sectors see as key to mitigating climate change. For example, plant-based diets require significantly fewer agricultural inputs, such as energy, petroleum, fertilisers, pesticides, herbicides and water, and emit far fewer greenhouse gases than do meat-heavy diets (74, 75). Further, while the focus on local and seasonal foods is shown to improve farmer-consumer relationships, increase revenue for small farmers, and encourage consumption of a wider diversity of foods, this is also a direct threat to the large volumes of food produced, processed and transported by the food industry (76). Both have the potential to negatively impact specific aspects of their industry and thus, the attitudes and behaviours of those involved (77).

The removal of the sustainability characteristics, “Promotion of plant-based diets” and “To limit red meat consumption as per recommendations” from future updates of NZ's FBDGs due to divergence of opinion and low levels of overall agreement, should not be considered from an environmental perspective. This is due to the disproportionate impact that agriculture sector emissions have been shown to have on the sustainability of NZ's food and health environment (71).

This divergence simply highlights the urgency for NZ to address these areas, further emphasising why any disproportionate influence the agriculture sector has in policy development may prevent NZ reaching its 2030 emission targets.

An urgent, multi-sectoral approach will be required during the development and implementation of sustainability guidelines to further investigate how these characteristics can be modified or communicated for effective inclusion (78, 79).

Only one characteristic, the recommendation “to purchase and support organic food produce,” received a low level of support for its inclusion in the EAGs from all three sectors: agriculture (21.8%), environment (59.7%), and health (33.9%). The low levels of agreement suggest that implementation of this characteristic might be problematic in NZ. However, unlike “red meat” and “plant-based,” knowledge regarding this characteristic, specifically the extent of agricultural chemical use, is low in NZ. More knowledge and awareness of impacts is required before successful inclusion. For example, recent international analyses examining the comparative impacts of organic and conventional systems have, of necessity, been limited to a few environmental indicators or in statistical strength of their inferences because of small sample size (80, 81). Also, in NZ, exposure to agricultural chemicals and contaminants from food as determined by Total Diet Study methodology is categorised as low (82).

Lastly, this study found that although only 28% of respondents believe that NZ's current food system is sustainable, this varied by sector. Over half (57%) of the agriculture sector respondents believe NZ's food system is sustainable, compared to <15% of respondents from the health and environmental sectors (*P* < 0.05). Further, agreement with this statement correlates with disagreement with the inclusion of seven of the individual sustainability characteristics. This opinion, held predominantly by the agriculture sector respondents, may explain the divergence of level of agreement from the agriculture sector towards these individual sustainability characteristics.

It further highlights the persistence of the academic landscape of sustainability, science, and education to consist of rather separate clusters of individual disciplines. Research regarding the current, largely unsustainable state of NZ's food system may not have been communicated in a way in which all stakeholders can recognise and relate to their sector. There had been an historic battle of understanding and defining the term “sustainability” across professions (83–86). Therefore, an opportunity for multi-disciplinary education may be a tool to bridge the gap between sectoral opinions and subsequent agreement with sustainability guidelines (62, 87). For example, in Sweden, although initially critical, the dairy organisations eventually expressed their support for the incorporation of environmental sustainability in the Swedish FBDGs once they became more informed, which then led to their engagement in the development of the guidelines.

A statistically significant positive correlation was observed between respondents' familiarity with the 2015 *Eating and Activity Guidelines for New Zealand Adults* and the individual sustainability characteristics. Similarly, a significant difference was found between the sector groups with 14% agriculture, 35% environment, and 91% health sector respondents being familiar with the guidelines. It is understandable that professionals in the environment and agriculture sectors are less familiar with the guidelines, compared to the health sector, as they are not frequently required in their work. However, it highlights that awareness and knowledge of the guidelines impacts agreement with inclusion of sustainability characteristics within the guidelines. This further highlights a need for dialogue among stakeholders from across the different sectors for successful FBDG development frameworks to exist and suggests where informative techniques may be useful (57).

A negative correlation was observed between respondents' agreement that “The World's current food system is sustainable” and “NZ's current food system is sustainable” with the agreement towards the inclusion of individual sustainability characteristics. This suggests that, the more unsustainable an individual believes a system is, the more likely they are to support the inclusion of sustainability characteristics into FBDGs. As suggested by the planned-behaviour theoretical framework, this may suggest an area where informational strategies can be aimed to inform attitudes and subsequent opinions (88, 89). Informational strategies may be aimed to increase sectoral professionals' knowledge of the unsustainable nature of the World's and New Zealand's food systems so as to heighten their awareness and increase agreement with policy change.

A positive correlation was observed between respondents' agreement with “New Zealand needs to adopt more/better agro-ecological farming practices” and seven of the individual sustainability characteristics, excluding “To purchase and support organic food produce” (P < 0.01). In contrast, a negative correlation was observed between respondents' agreement with “Sustainability recommendations should be included in the “Eating and Activity Guidelines for Adults” and all eight of the sustainability characteristics. This suggests discrepancy between the guidelines as a whole and the individual characteristics of a sustainable diet. This highlights the need for characteristics to be treated individually, with input gathered from, and education provided to, all stakeholders.

### Strengths and Limitations

A strength of this study is that it provides a snapshot of how the inclusion of sustainability characteristics within dietary guidelines may be received in NZ at this time. Despite a myriad of evidence calling for multi-sectoral approaches to guideline development, this study has provided the first detailed examination of the degree of convergence between professional sectoral groups, both internationally and in NZ, for the inclusion of sustainability characteristics into dietary guidelines (78, 79). There are, of course, limitations to this study. Self-reported responses to the attitudinal questions may be influenced by social desirability, a sense of social responsibility as a result of increasing global awareness of sustainability and its importance (90). Respondents may have answered the questions in a way they believe they should or wish to be perceived as a result of their knowledge on the subject, either intentionally or unintentionally (91). However, the results of this study, including several areas with little or no convergence of opinion, suggest this did not impact several responses.

## Conclusion

The high level of support for sustainability integration into the EAGs found in this study highlights the degree to which sectors view the importance of sustainable food systems in NZ. This research also highlights that, as demonstrated in America, Australia, and Sweden, NZ is at risk of disunity between sectors, leading to opposition and subsequent abandonment of sustainability characteristics within EAGs following implementation. Although there is currently an apparent disconnect between the health, agricultural, and environmental sector respondents' opinions and attitudes, there are also undeniable links which present unexplored opportunities for cooperative problem solving. NZ is in a unique position for, prior to implementation, multi-sectoral participatory dialogue to identify and clarify the specific shape that sustainability should take in FBDGs. The significantly lower level of support from the agriculture sector respondents in this study highlights the need to mitigate the risk of predominantly agriculture sectoral lobbying in NZ via wider, earlier and higher level of engagement with relevant sectors and individuals to increase overall multi-sectoral agreement (60, 61). Instead of excluding the primary industries from FBDG development and implementation, several changes, namely, to enhance transparency and to manage biases and conflicts of interest, may be adopted prior to implementation (63). Overall, this research has brought together a diverse range of academic and professional expertise that spans the agriculture, environment, and health sectors. Convergences and divergences of opinions of these sectors have been evaluated. The findings of this study should be of interest to government sectors that can influence sustainability and health, for example, departments or ministries of health, education, primary industries, regional development, agriculture, food, and finance.

## Data Availability

The raw data supporting the conclusions of this manuscript will be made available by the authors, without undue reservation, to any qualified researcher.

## Ethics Statement

This project was evaluated by the Massey University Human Ethics Committee and judged to be low risk. Consequently, it has not been reviewed by one of the University's Human Ethics Committees. The researcher(s) named in this document are responsible for the ethical conduct of this research.

## Author Contributions

RJ was responsible for the research proposal, literature review, ethics application, study design, questionnaire development, liaising with key stakeholders, data collection, statistical analysis, and preparing the final manuscript. BB and CW assisted with the research question, study design and questionnaire development, provided critical feedback and helped shape the research and analysis. All authors discussed the results and assisted with the editing, finalizing and submission of the final manuscript.

### Conflict of Interest Statement

The authors declare that the research was conducted in the absence of any commercial or financial relationships that could be construed as a potential conflict of interest.
