# Supplementary material for: New Zealand's Food System Is Unsustainable: A Survey of the Divergent Attitudes of Agriculture, Environment, and Health Sector Professionals Towards Eating Guidelines
Source: Front Nutr. 2019 Jul 16;6:99. doi: 10.3389/fnut.2019.00099 (PMC6648584; doi:10.3389/fnut.2019.00099)
Supplement: Supplementary file 1 [file Table_3.DOCX]

# Appendices

Appendix A: Questionnaire

1. What is your gender?

Male

Female

Other

2. What is your age?

18 to 24

25 to 34

35 to 44

45 to 54

55 to 64

65 to 74

75 or older

3. What is the highest level of education you have completed (or are currently completing)?

No qualification

High school

Bachelor’s Degree

Post-graduate and honours degrees

Overseas secondary school qualification

4. Which professional sector do you identify with?

Environmental

Agriculture - Meat, Dairy, Seafood and Aquaculture

Health - Private, NGOs, DHBs

Other (please specify)

5. Within this sector, which sub-sector do you identify with?

Agriculture - Meat

Agriculture - Dairy

Agriculture - Seafood and aquaculture

Agriculture - Horticulture

Health - Nutrition

Health - Other

Environment

Other (please specify)

6. Please state whether you agree or disagree with the following statements:

|  | Strongly Agree | Agree | Don't know | Disagree | Strongly Disagree |
| --- | --- | --- | --- | --- | --- |
| The world’s current food system is sustainable |  |  |  |  |  |
| New Zealand's current food system is sustainable |  |  |  |  |  |
| New Zealand needs to adopt more/better agro-ecological farming practices |  |  |  |  |  |
| I am familiar with the 2015 'Eating and Activity Guidelines for New Zealand Adults' |  |  |  |  |  |
| Sustainability recommendations should be included in the 'Eating and Activity Guidelines for Adults' |  |  |  |  |  |

7. The following characteristics of a sustainable diet should be included and linked to both human and environmental health in the ‘Eating and Activity Guidelines for New Zealand Adults’:

|  | Strongly agree | Agree | Don't Know | Disagree | Strongly Disagree |
| --- | --- | --- | --- | --- | --- |
| Promotion of diet diversity/variety of whole foods |  |  |  |  |  |
| Promotion of plant-based diets |  |  |  |  |  |
| To limit red meat consumption as per recommendations |  |  |  |  |  |
| To limit processed meat consumption as per recommendations |  |  |  |  |  |
| To consume recommended serves of dairy products |  |  |  |  |  |
| Promotion of sustainable seafood consumption |  |  |  |  |  |
| To limit/reduce ALL processed foods high in fat, salt and sugar as per recommendations |  |  |  |  |  |
| To purchase and support local food produce |  |  |  |  |  |
| To purchase and support seasonal food produce |  |  |  |  |  |
| To purchase and support organic food produce |  |  |  |  |  |
| Standards for the ethical treatment of livestock |  |  |  |  |  |
| To reduce food waste |  |  |  |  |  |
| Promotion of sustainable lifestyle behaviours (for example, physical activity) |  |  |  |  |  |

8. Please state your agreement with the following statements:

|  | Strongly Agree | Agree | Don't Know | Disagree | Strongly Disagree |
| --- | --- | --- | --- | --- | --- |
| I support country of origin labelling of foods |  |  |  |  |  |
| I support labelling foods with New Zealand Geographic Indicators (e.g. Keri Keri oranges, Pukekohe potatoes, Gimblett Gravels wine) |  |  |  |  |  |

Survey Complete!

If you would like to provide any additional comments or feedback please email R.Jones@massey.ac.nz

Appendix B: Table 1. Agreement to sustainability statements by gender, age and education level.

| **Sustainability statement** | **Gender collapsed (*P*-value)** | **Age collapsed**  **(*P*-value)** | **Education collapsed – Graduate degree**  **(*P*-value)** | **Education - high school** | **Education – postgraduate P** |
| --- | --- | --- | --- | --- | --- |
| The world’s current food system is sustainable | 0.00* | 0.028* | 0.000* | 0.00* | 0.017* |
| New Zealand's current food system is sustainable | 0.001* | 0.227 | 0.024* | 0.024* | 0.000* |
| New Zealand needs to adopt more/better agro-ecological farming practices | 0.043* | 0.424 | 0.546 | 0.702 | 0.174 |
| I am familiar with the 2015 “Eating and Activity Guidelines for New Zealand Adults” | 0.000* | 0.508 | 0.004* | 0.053* | 0.000* |
| Sustainability recommendations should be included in the “Eating and Activity Guidelines for Adults” | 0.065 | 0.121 | 0.410 | 0.483 | 0.320 |
| **Sustainability characteristic** | **Gender collapsed (*P*-value)** | **Age collapsed**  **(*P*-value)** | **Education collapsed – Graduate degree**  **(*P*-value)** | **Education - high school** | **Education – postgraduate P** |
| Promotion of diet diversity/variety of whole foods | 0.909 | 0.515 | 0.349 | 0.656 | 0.979 |
| Promotion of plant-based diets | 0.003* | 0.075 | 0.000* | 0.000* | 0.000* |
| To limit red meat consumption as per recommendations | 0.000* | 0.087 | 0.000* | 0.000* | 0.000* |
| To limit processed meat consumption as per recommendations | 0.001* | 0.099 | 0.000* | 0.001* | 0.001* |
| To consume recommended serves of dairy products | 0.163 | 0.878 | 0.780 | 0.866 | 0.553 |
| Promotion of sustainable seafood consumption | 0.348 | 0.670 | 0.904 | 0.873 | 0.830 |
| To limit/reduce ALL processed foods high in fat, salt and sugar as per recommendations | 0.000* | 0.263 | 0.312 | 0.492 | 0.814 |
| To purchase and support local food produce | 0.005* | 0.839 | 0.074 | 0.127 | 0.740 |
| To purchase and support seasonal food produce | 0.016 | 0.873 | 0.120 | 0.313 | 0.803 |
| To purchase and support organic food produce | 0.012* | 0.703 | 0.626 | 0.895 | 0.574 |
| Standards for the ethical treatment of livestock | 0.013* | 0.186 | 0.187 | 0.445 | 0.030* |
| To reduce food waste | 0.869 | 0.452 | 0.228 | 0.498 | 0.615 |
| Promotion of sustainable lifestyle behaviours (for example, physical activity) | 0.861 | 0.538 | 0.874 | 0.961 | 0.982 |
| **Sustainability characteristic** | **Gender collapsed (*P*-value)** | **Age collapsed**  **(*P*-value)** | **Education collapsed – Graduate degree**  **(*P*-value)** | **Education - high school** | **Education – postgraduate P** |
| I support country of origin labelling of foods | 0.051 | 0.970 | 0.153 | 0.376 | 0.922 |
| I support labelling foods with New Zealand Geographic Indicators | 0.318 | 0.176 | 0.737 | 0.884 | 0.744 |

* identifies level of significance *P* < 0.05

Two variables were created for ‘Education’; High School 1=Yes 0=No and Postgrad 1=Yes  0=No. No variable was created for Bachelors as that is the reference category. An ANCOVA was then run with covariates; gender, age, high school and postgrad and the fixed factor was sector. The dependent variable was the item score of each statement e.g. I support labelling foods with New Zealand Geographic Indicators.

Results show that there is a statistically significant difference between gender groups and level of agreement as determined by one-way ANOVA; “The world’s current food system is sustainable”, “New Zealand's current food system is sustainable”, “New Zealand needs to adopt more/better agro-ecological farming practices”, “I am familiar with the 2015 “Eating and Activity Guidelines for New Zealand Adults”, “Promotion of plant-based diets”, “To limit red meat consumption as per recommendations”, “To limit processed meat consumption as per recommendations”. There is a statistically significant difference between age groups and level of agreement as determined by one-way ANOVA; “The world’s current food system is sustainable”, “To limit red meat consumption as per recommendations”, “I support labelling foods with New Zealand Geographic Indicators”. There is a statistically significant difference between education groups and level of agreement as determined by one-way ANOVA; “The world’s current food system is sustainable”, “New Zealand's current food system is sustainable”, “I am familiar with the 2015 “Eating and Activity Guidelines for New Zealand Adults”, “Promotion of plant-based diets”, “To limit red meat consumption as per recommendations”, “To limit processed meat consumption as per recommendations”. Therefore, ANCOVAs were run to detect a difference in means of the sector levels of agreements whilst controlling for covariates.
